# Comparison of ultrasound and MRI shows equivalent accuracy and reliability in acromial index measurement

**DOI:** 10.1038/s41598-025-07370-2

**Published:** 2025-07-03

**Authors:** Christian T. Schamberger, Tobias Grossner, Christian Fischer, Sebastian Findeisen, Thomas Ferbert, Jessica C. Boepple, Arnold J. Höppchen, Gerhard Schmidmaier, Stephan Stein

**Affiliations:** 1 Clinic for Trauma- and Reconstructive Surgery, University Clinic Heidelberg, Schlierbacher Landstr. 200A, 69120 Heidelberg, Germany; 2 Arcus Sports Clinic, Pforzheim, Germany; 3 Neckar-Odenwald-Hospital Mosbach, Academic Teaching Hospital of Heidelberg University, Heidelberg, Germany; 4 Medical Faculty Mannheim, University Heidelberg, Heidelberg, Germany

**Keywords:** Acromial index, Ultrasound imaging, Rotator cuff pathology, Shoulder diagnostics, Clinical trial design, Diseases

## Abstract

The acromial index, a radiological parameter assessing the subacromial space, is vital for diagnosing conditions like rotator cuff tears and subacromial impingement syndrome as the acromial Index influences the choice of therapy. Traditionally determined via X-ray, AI is increasingly assessed using MRI due to its superior image quality. However, MRI’s cost and limited availability highlight the need for alternative methods. Ultrasound offers a promising, cost-effective alternative, providing real-time imaging without radiation exposure. This study aims to compare the accuracy and reliability of ultrasound-based AI determination with MRI. This retrospective study enrolled patients with shoulder complaints who underwent both MRI and ultrasound examinations between November 2021 and October 2022. Inclusion criteria included appropriate MRI and ultrasound images of the shoulder joint. AI was measured on MRI images and a modified AI was assessed using ultrasound. Statistical analyses evaluated the accuracy and reliability of both methods, focusing on intra- and interobserver consistency. A total of 113 patients (53.2 years average age, 31.3% female) were included. The mean MRI-determined AI was 0.65 (SD 0.065), with 15.6% showing pathological AI. Ultrasound-determined modified acromial index averaged 0.74 (SD 0.122), with 33% showing pathological modified acromial index. Comparative analysis showed no significant difference between MRI and ultrasound measurements (*p* = 0.237). Both methods demonstrated high intra- and interobserver reliability (ICC values > 0.9). Ultrasound-assisted AI determination is a valid, reproducible, and reliable alternative to MRI. Its advantages include cost-effectiveness, accessibility, and absence of ionizing radiation, making it particularly useful in resource-limited settings.

## Introduction

The precise imaging diagnosis and assessment of shoulder disorders is of great importance for the planning and implementation of therapeutic measures. The acromial index (AI), a radiological parameter for assessing the subacromial space or the lateral roofing of the humeral head by the acromion, plays a decisive role in the diagnosis of pathological changes such as rotator cuff tears, and particularly subacromial impingement syndrome^1–3^. The AI describes the relationship between the humeral head and the lateral canopy through the acromion and provides important information about potential compression of the rotator cuff in this area^1,4^. This morphometric relevance is further supported by studies investigating the shape and structure of the acromion in symptomatic patients. Koca et al.^5^ found significant associations between acromion types and the presence of shoulder pain, emphasizing the importance of detailed anatomical evaluation in the context of rotator cuff-related disorders. Nyffeler et al. defined AI values higher than 0.7 to be associated with rotator cuff tears (RCT)^1^. Morelli et al. found in their meta-analysis that patients with a larger acromial index have a greater likelihood for non-traumatic RTC tears^6^.

Traditionally, AI is determined using native X-ray diagnostics^1^. MRI is considered the gold standard in diagnosing pathologies of the shoulder due to its high image quality and detailed visualization of soft tissue and bone structures^7,8^. It enables precise assessment of the subacromial space and adjacent tissue and is therefore a key diagnostic method in orthopedics and trauma surgery and has been proven to be as accurate as the X-ray in determining the AI^9^. However, MRI is cost-intensive, time-consuming, and not always immediately available in clinical practice. In addition, performing an MRI requires special infrastructure and trained personnel, which makes access to this diagnostic method difficult, especially in rural or resource-poor regions^10^. Determining the AI by X-ray is a cheap and quick alternative, but has the disadvantages of a not-radiation-free examination and can also only show the bony structures of the shoulder missing the visualization of the soft-tissue like the rotator-cuff, labrum et cetera^11,12^. Furthermore, sufficient true a.p.-view is needed for correct assessment of qualitative values of the shoulder joint especially in context of subacromial impingement and rotator cuff tears^13–15^.

In recent decades, ultrasound has established itself as a promising, cost-effective, and quick alternative procedure for assessing the shoulder joint^16,17^. Ultrasound offers the advantage of a dynamic examination in which both static and functional information about the shoulder can be obtained^18^. The method is widely used, relatively easy to perform and does not require complex technical equipment. Ultrasound also enables real-time imaging, which is particularly useful when examining movement-dependent complaints^16,18,19^.

Despite these advantages, there is still uncertainty regarding the accuracy and reliability of the ultrasound-based procedure compared to MRI. To date, no studies have been conducted on the accuracy of determining the AI using sonography, although other sonographic supported parameters have already been validated in the area of the shoulder joint^16,20,21^.

The knowledge gained should help to expand the diagnostic possibilities in shoulder orthopedics and optimize the use of ultrasound in routine clinical practice. This could be particularly important in situations where access to MRI is limited. In conclusion, this study should not only strengthen the scientific basis for ultrasound-guided determination of AI, but also provide practical impulses for improving patient care.

The aim of this study is to systematically compare the ultrasound-based determination of the acromial index with the MRI-based method. Both the measurement accuracy and the clinical applicability of the two methods will be compared. A particular focus is on the evaluation of intra- and interobserver reliability in order to determine the consistency of the measurements between different examiners and with repeated measurements by the same examiner.

## Materials and methology

### Study design and patient recruitment

This retrospective study was conducted following the guidelines outlined in the “Strengthening the Reporting of Observational Studies in Epidemiology (STROBE) Statement” for observational study reporting^22^.

This study included patients presenting with shoulder-related complaints, who underwent both magnetic resonance imaging (MRI) and ultrasound examinations of the affected shoulder joint from November 2021 to October 2022. The objective was to systematically review and compare the archived MRI findings with the corresponding ultrasound results.

The study adhered to the principles of the Declaration of Helsinki and received approval from the Ethics Committee of the University of Heidelberg (S-025/2024) on February 19, 2024. Informed consent for all illustrations included in the manuscript was obtained from the respective individuals.

### Inclusion and exclusion criteria

Exclusion and inclusion criteria were established to ensure the study’s reliability and precision. The exclusion criteria encompassed conditions such as advanced glenohumeral osteoarthritis (Kellgren–Lawrence grade > II), indications of humeral head necrosis, open epiphyseal plates, recent or healed fractures involving the humeral head or glenoid, disruptions in the acromioclavicular joint, previous shoulder joint total endoprosthesis, and technically inadequate MRI or ultrasound examinations.

We performed MRI assessments using standardized coronal, sagittal, and axial planes with T1 or TSE weighting without contrast enhancement. Within the coronal plane, the essential parameter AI was measured.

Inclusion criteria were applied to enhance study validity. For inclusion, a valid MRI examination in the coronal plane with T1 and/or TSE weighting was required. In addition, a corresponding sonographic examination of the same shoulder joint should be carried out at an early stage, based on the guidelines of the “German Society for Ultrasound in Medicine” (DEGUM)^18^. Finally, only patients reporting symptoms localized to the shoulder joint were selected for participation in the study.

### Ultrasound-assisted assessment of the mAX

A standard ultrasound device with a linear transducer operating at a variable frequency range of 7–15 MHz was used for the sonographic examinations.

**Fig. 1:**
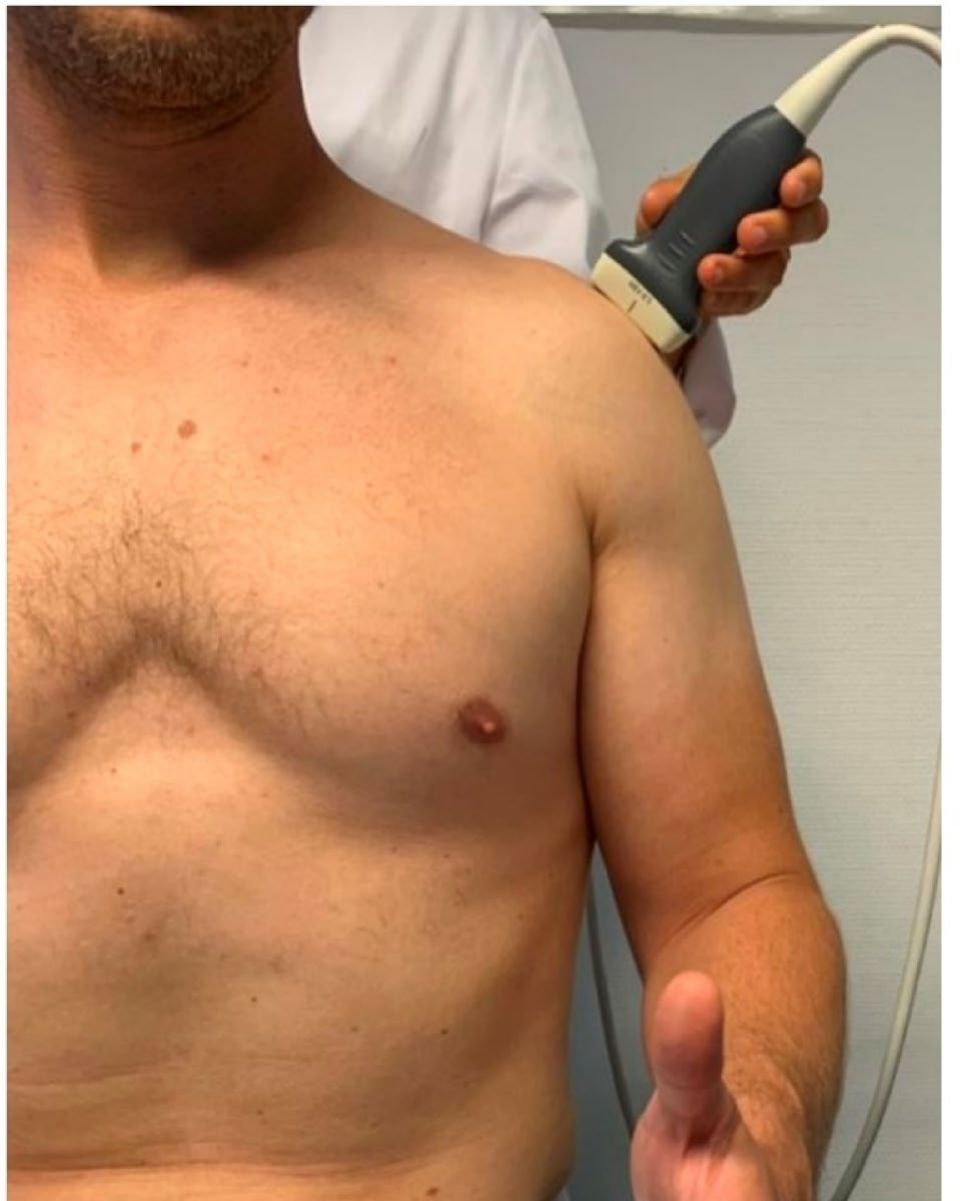
Position of the ultrasound-transducer in the lateral longitudinal section. This figure shows the correct placement of the linear ultrasound transducer in the lateral longitudinal section of the shoulder. The transducer is oriented to visualize the lateral acromion and the spherical contour of the humeral head, enabling accurate identification of anatomical landmarks necessary for calculating the modified acromial index (mAX). This positioning allows standardized and reproducible acquisition in accordance with DEGUM recommendations.

**Fig. 2:**
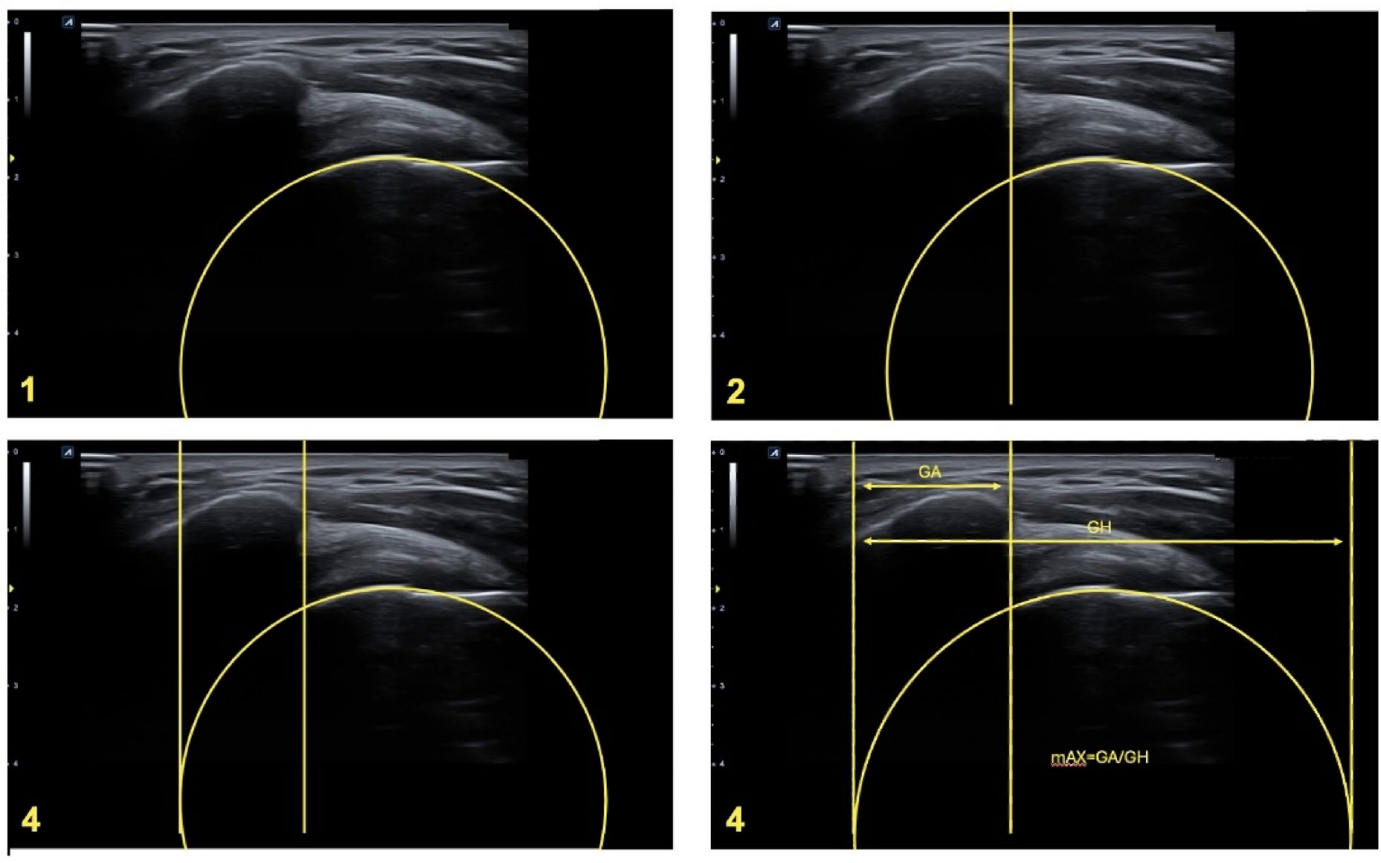
Step-by-step determination of the ultrasound assisted mAX. The image series illustrates the sonographic procedure used to determine the mAX. After identifying the relevant anatomical landmarks (lateral acromion and humeral head), a digital circle was fitted to the spherical contour of the humeral head. Vertical reference lines were drawn tangential to the lateral acromion, the medial surrogate for the glenoid fossa, and the lateral edge of the humeral head. The modified acromial index was calculated as the ratio between the distances from the medial surrogate to the acromion (GA) and to the humeral head (GH). This method enables an approximation of the traditional AI using real-time ultrasound imaging.

1. The examination was carried out in a sitting position, with the examined arm maintained in a neutral position with no abduction and the circular structure of the humeral head included in the imaging (Fig. 1).
2. To accurately determine the modified acromial index (mAX), the lateral aspect of the acromion and the spherical shape of the humeral head were visualized in the superior longitudinal section, with all relevant anatomical structures clearly identified.
3. Afterwards, we expanded the sonographically visualized circular section of the humeral head to a complete circle (Fig. 2(1)) using an image viewing, editing, and measuring program (Centricity, Universal Viewer Zero Footprint Client, Version: 6.0 SP11.2.3).
4. Next, we positioned a first line vertically, perpendicular to the monitor’s horizontal edges, and tangential to the lateral edge of the acromion (AE) (Fig. 2(2)).
5. A second vertical line was positioned tangentially to the medial contour of virtually visualized humeral head represented by the circle. This point was used as a pragmatic surrogate for the glenoid fossa (GF), acknowledging that direct visualization of the glenoid is not feasible due to acoustic limitations (Fig. 2(3)).
6. We drew a third line tangential to the lateral aspect of the humeral head, parallel to the first and second lines (HH) (Fig. 2(4)).
7. Finally, we measured the distances between the surrogate GF and AE (GA) and between the surrogate GF and HH (GH), and calculated the ratio (Fig. 2(4)).

### MRI-assisted assessment of the AI

**Fig. 3:**
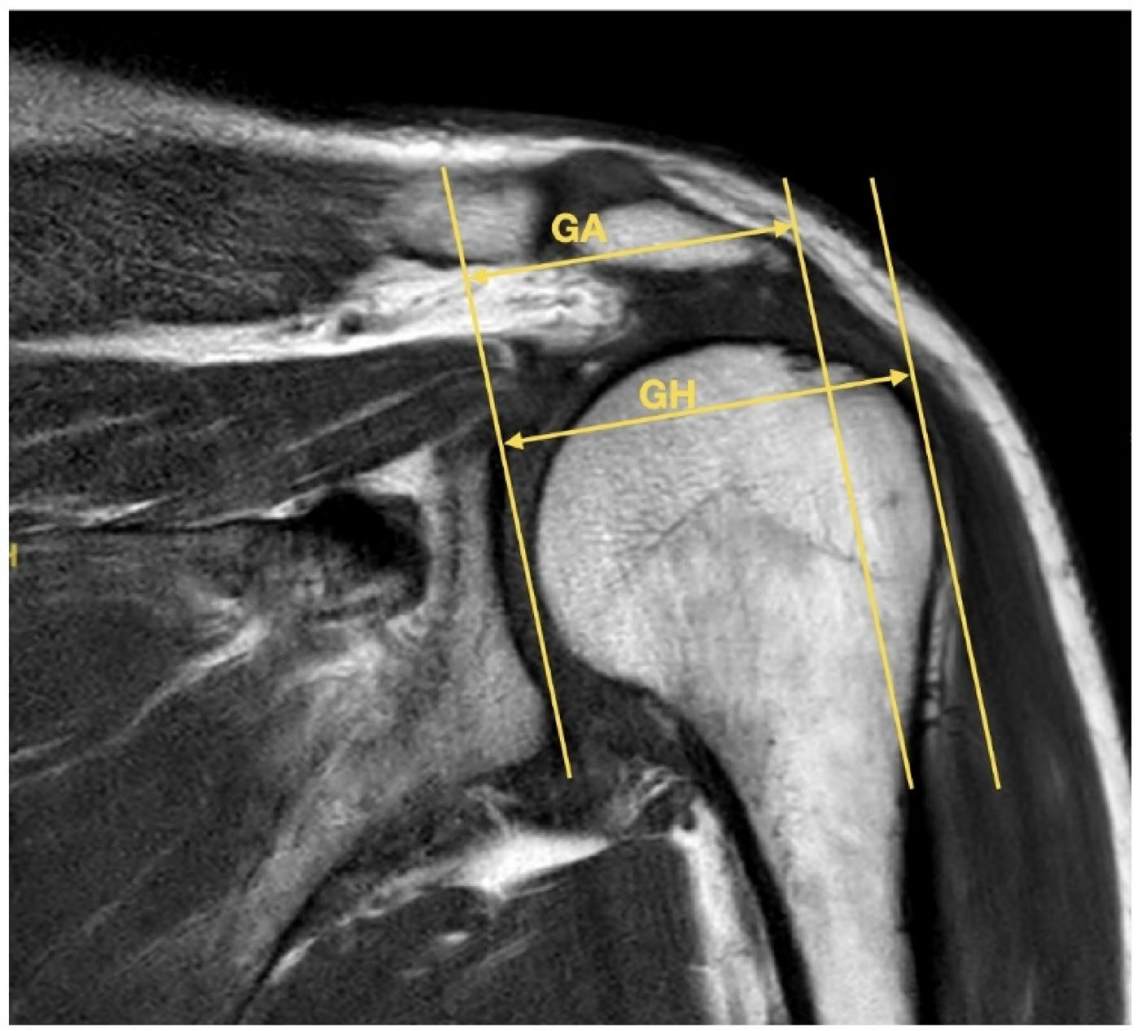
MRI assisted determination of the acromial index. This figure displays the method used to calculate the standard acromial index (AI) from MRI scans. Measurements were taken in the coronal plane, using the distance from the glenoid cavity to the lateral acromion and the distance to the lateral humeral head margin. The ratio of these two distances defines the AI. MRI offers high-resolution anatomical visualization, enabling precise identification of these landmarks in a static imaging context.

The AI measurement based on MRI was conducted using native MRI, utilizing coronal planes for quantitative assessment. The AI was calculated as the ratio between the distance from the glenoid cavity to the lateral edge of the acromion and the distance from the glenoid cavity to the lateral edge of the humerus, as illustrated in Fig. 3.

### Statistical analysis

To ensure the study’s reliability and objectivity, two DEGUM-certified examiners independently evaluated both MRI and ultrasound images in accordance with society guidelines. Each examiner conducted three measurements on the ultrasound images and three on the MRI images, resulting in a total of twelve measurements per joint.

For the statistical analysis, we used SPSS software, version 27 (IBM SPSS Statistics for iOS, Armonk, NY, USA). Normality of continuous variables was tested using the Kolmogorov–Smirnov test. We measured inter- and intra-observer reliability using intraclass coefficients (ICC) and interpreted the results using the guidelines set by Landis and Koch^23^. A value below 0.01 indicates poor agreement; between 0.01 and 0.20, slight agreement; 0.21–0.40, fair agreement; 0.41–0.60, moderate agreement; 0.61–0.80, substantial agreement; and 0.81–1.00, almost perfect agreement.

The significance level was set at *p* ≤ 0.05. To achieve a robust effect size of η^2^ = 0.14 with a power of 0.9, a priori, a total of 103 participants were calculated for a meaningful outcome, and α was set to 0.05^24^. We plotted the results of both methods into a Bland–Altman diagram for graphical comparison and analysis^24^. Interrater reliability was assessed, with a value of 0.7 or higher being considered satisfactory^25^.

## Results

### Demography

A total of 115 patients (73/63.5% left and 42/36.5% right shoulder joints), with an average age of 53.2 years (range 18–79 years; 36/31.3% female and 79/68.7% male), met the inclusion criteria and were enrolled in the study (Table 1).

**Table 1:** Demographic data of the study cohort.

	N	Percent	Mean	SD
Sex				
Male	79	68.7		
Female	36	31.3		
Side				
Left	73	63.5		
Right	42	36.5		
Age (years)				
Mean			53.15	13.65

The table summarizes the baseline characteristics of all included patients (n = 115). Variables include sex, side of the examined shoulder (left/right), and age distribution. The majority of patients were male (68.7%) and presented with left-sided complaints (63.5%). The mean age was 53.2 years (SD = 13.65), with a wide age range (18–79 years), ensuring representation of a broad adult population.

### MRI-assisted assessment of the AI

In the MRI scans, we found a mean AI of 0.65 (SD 0.065). 18 patients (15.6%) exhibited a pathological AI, 97 patients (84.3%) showed normal AI. The gender distribution for the cohort with an AI > 0.7 comprised 12 males (66.7%) and 6 females (33.3%), while the cohort with a normal AI consisted of 67 males (69.1%) and 30 females (30.9%). Two minor outliers were identified during the data analysis and excluded from the subsequent statistical analyses; thus, 113 patients underwent an ultrasound-assisted assessment of the mAX as well as further statistical analysis (Table 2).

**Table 2:** MRI assisted Assessment of the AI.

	N	Percent	Mean	SD
AI in MRI				
AI			0.65	0.065
Pathological AI	18	15.6		
Male	12	66.7		
Female	6	33.3		
Normal AI	97	84.3		
Male	67	69.1		
Female	30	30.9		

This table presents the distribution of standard acromial index (AI) values measured via MRI. The overall mean AI was 0.65 (SD = 0.065). Using a pathological threshold of > 0.7, 18 patients (15.6%) were classified as pathological. Gender distribution is provided for both normal and pathological AI groups. MRI served as the reference imaging modality in this comparative study.

### Ultrasound-assisted assessment of AI (mAX)

The ultrasound examination of the mAX showed a mean AI of 0.74 (SD 0.122). 38 patients (33%) showed a pathological mAX over 0.7, 77 patients (67%) showed normal mAX. In the cohort over 0.7 26 (68.4%) were male, 12 (31.6%) were female. 53 (68.8%) patients with a normal mAX were male while 24 (31.2%) were female (Table 3).

**Table 3:** Ultrasound assisted Assesment of the modified AI (mAX).

	N	Percent	Mean	SD
mAX in US				
mAX			0.74	0.122
Pathological mAX	38	33		
Male	26	68.4		
Female	12	31.6		
Normal mAX	77	67		
Male	53	68.8		
Female	24	31.2		

This table details the results of the mAX measurements obtained via ultrasound in 113 patients. The mean mAX was 0.74 (SD = 0.122). Applying the same threshold of > 0.7 as used in MRI-based AI, 38 patients (33%) were categorized as pathological. The table includes gender distribution within pathological and normal subgroups. These findings highlight a higher detection rate of pathological indices with ultrasound, warranting further investigation into clinical relevance and threshold validity.

### Comparative analysis

**Fig. 4:**
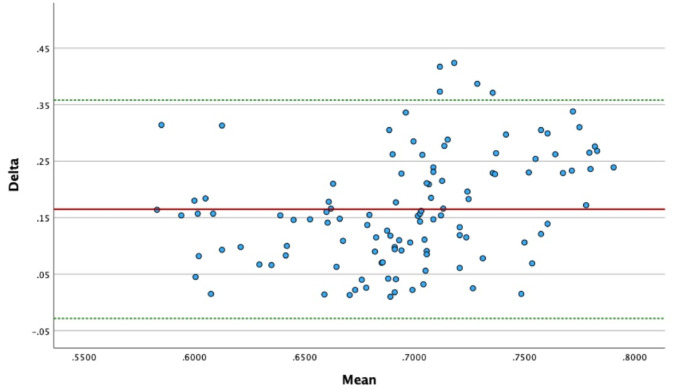
Bland–Altman-plot showing 5 outliers, red line = mean Delta of AI and mAX, green dashed lines = 1.96 * SD. The plot illustrates the agreement between the modified acromial index (mAX) measured via ultrasound and the standard acromial index (AI) measured via MRI. The X-axis shows the mean of both measurements, and the Y-axis indicates the difference between the two methods (mAX − AI). The mean difference is represented by a solid line, with dashed lines indicating the upper and lower limits of agreement (mean ± 1.96 SD). The majority of values fall within the limits of agreement, suggesting reasonable overall consistency between methods. Five data points lie outside these limits, indicating outliers and potential individual measurement deviations.

Both the AI (MRI) and the mAX (ultrasound) exhibited normal distributions based on the Shapiro–Wilk test (*p* > 0.05). The comparative statistical analysis of AI assessed via MRI and ultrasound assisted evaluation of the mAX using a Bland–Altman-plot showed good results with only five outliers (Fig. 4). This suggests that ultrasound may serve as a feasible alternative for acromial index (AI) estimation within this range.

**Fig. 5:**
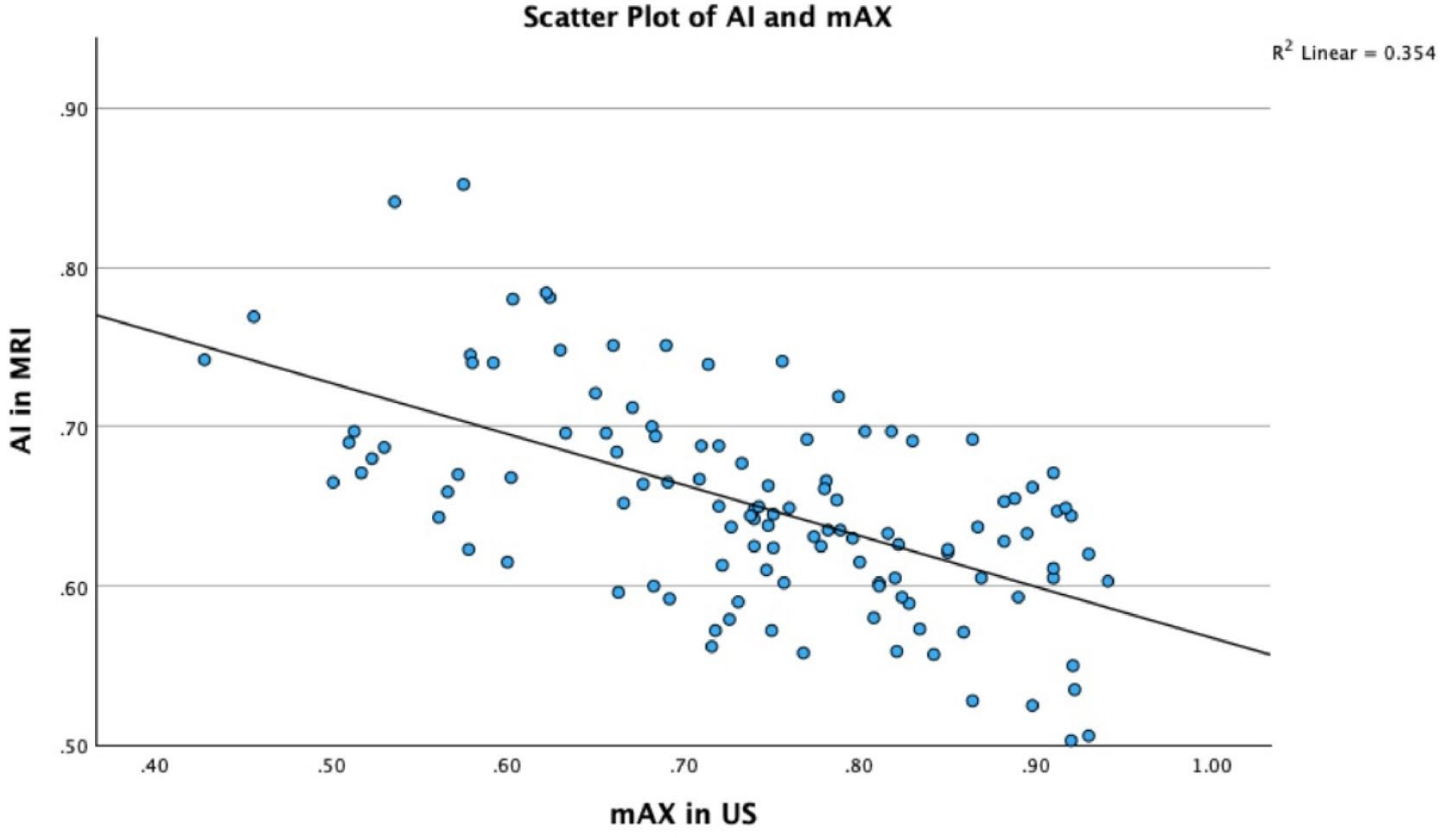
Scatter plot of AI and mAX with linear Regression, R^2^ = 0.354. This scatter plot displays the relationship between the ultrasound-derived modified acromial index (mAX) and the MRI-derived acromial index (AI). Each dot represents a single patient measurement. A linear regression line is included to visualize the correlation trend, with the shaded area indicating the 95% confidence interval. The Pearson correlation coefficient (r = − 0.595) demonstrates a moderate negative correlation, while the coefficient of determination (R^2^ = 0.354) indicates a weak linear agreement.

In contrast, the scatter plot analysis revealed a moderate negative correlation between mAX and MRI-derived AI (r = − 0.595; *p* < 0.001), and a low coefficient of determination (R^2^ = 0.354), indicating a weak and inconsistent linear relationship between the two modalities (Fig. 5). Despite the lack of a significant difference in mean values (*p* = 0.237), the observed variability and systematic divergence suggest that the two methods may not yield interchangeable results across the full spectrum of measured values.

Feasible values were observed for intra-observer reliability, regarding both mAX and AI measured either in MRI or ultrasound. Furthermore, it was found an excellent, almost perfect intra-observer agreement for both CSA and AI measurements made in MRI and ultrasound. There was near-perfect interobserver agreement for both mAX and AI measurements obtained via ultrasound, and substantial interobserver agreement for measurements made using MRI.

The ICC values for AI and mAX obtained from ultrasound and MRI for both observers were 0.9 and 0.92, respectively. The mean difference in AI values measured by ultrasound and MRI was 0.14 for observer 1 and 0.3 for observer 2. The mean difference in mAX values measured by ultrasound and MRI was 0.1 for observer 1 and 0.21 for observer 2.

## Discussion

To the best of the authors knowledge, the results of this study demonstrate for the first time that sonographic assisted determination of the modified acromial index is a safe, reproducible and valid method compared to the MRI-based determination of the standard acromial index. No significant difference was found between the two examination methods.

Factors like ionizing radiation in X-Ray and CT as well as high costs and time consuming examinations in MRI highlight the importance of ultrasound as a complementary imaging technique.

If the acromial index is incorrectly determined, this may lead to a misjudgment of the pathology, which in turn affects the selection of the appropriate therapy. Especially when considering surgical interventions, such as subacromial decompression, an accurate determination of the Acromial Index is crucial to avoid unnecessary or even harmful operations.

In their study, Schiefer et al. describe comparable values for the acromial index in both MRI and standard X-ray imaging^9^. In everyday clinical practice, it is often difficult to obtain a correct antero-posterior X-ray of the shoulder joint, which can have a negative effect on the measurement of the AI, as the specified measurement points on both the glenoid and the lateral acromion edge cannot be determined correctly. This can occur by malrotation of the arm or an incorrect positioning of the scapula^13^.

In addition to conventional X-rays, CT is considered a valid imaging method for determining the AI^9^, although here too, even more than with X-rays, the radiation exposure for the patient must be mentioned as a disadvantage of this method. Furthermore, in cross-sectional imaging, however, the same problem exists as with conventional X-rays, as the most lateral extension of the humeral head does not always lie correctly in the coronal measurement plane due to the arm position, however in ultrasound this problem does not exist due to the live imaging modality.

The advantage of sonographic determination of the acromial index is the dynamic examination, so that the greater tuberosity can be visualized sonographically in its maximum extent in order to increase the accuracy of measurement. The humeral retroversion varies between 10° and 40° so that the arm is either rotated accordingly for the measurement or the transducer is positioned to the optimum setting^13^. This is an advantage that is reserved exclusively for ultrasound examinations in this radiation-free form.

In ultrasound both soft tissue structures and bony structures can be evaluated.

Ultrasound training is usually part of specialist training in orthopedic and trauma surgery, so that ultrasound diagnostics is used regularly on a daily basis both in clinics and in the outpatient sector.

Compared to previous studies assessing AI, demographic data from the present study are comparable^9^. The ICC values for intra-observer agreement exceeded those for inter-observer agreement, indicating that individual observers were more consistent in their measurements over time than when compared to each other. Intra-observer agreement was slightly higher for MRI than for ultrasound. Despite this, the inter-observer agreement remained high, indicating substantial consistency between different observers. Furthermore, there was a high correlation between methods, with similar absolute AI and mAX values observed in both ultrasound and MRI, resulting in high ICC values. These results suggest that both MRI and ultrasound are equally effective for these measurements.

However mAX revealed 33% pathological AIs compared to 15.6% in MRI. This can be attributed to the fact that ultrasound always captures the maximum expression of the bony structures, whereas in MRI, these structures might lie between the slices.

The cut-off value of 0.7 used to classify a pathological mAX was derived analogously from the established threshold described by Nyffeler et al. for radiographic and MRI-based Acromial Index assessments^1^. This value has been frequently cited in the literature and serves as a widely accepted reference point for identifying an increased risk of rotator cuff pathology. However, the direct transfer of this threshold to sonographic measurements must be interpreted cautiously. Furthermore, the higher prevalence of mAX values above 0.7 in our cohort (33%) compared to MRI-based AI (15.6%) raises the question of whether this threshold might lead to overdiagnosis when applied without adjustment. In light of this, we acknowledge that the use of the same pathological cut-off value across both modalities requires further validation. Future research, ideally involving prospective, multicenter studies, is necessary to determine a clinically appropriate and sonography-specific threshold for mAX that balances sensitivity and specificity. Independent external validation would be essential to substantiate this adaptation and confirm its diagnostic relevance.

Until such data are available, the threshold of 0.7 for mAX should be regarded as a provisional reference value that enables comparability with existing MRI and radiographic literature but may not represent the definitive diagnostic criterion for ultrasound-based AI evaluation.

While advanced MRI protocols utilizing thin slices and multiplanar reconstructions can substantially reduce the risk of missing key anatomical landmarks, thereby enabling highly reproducible and accurate measurements, it is important to note that such protocols are not routinely implemented in standard clinical settings. In everyday practice, particularly in non-specialized or resource-constrained environments, examinations are frequently conducted using conventional protocols that may lack high-resolution slice thickness or multiplanar capabilities. These limitations are often dictated by time constraints, scanner availability, and economic considerations. Consequently, although MRI offers a theoretically superior reproducibility, ultrasound may present a pragmatic advantage in clinical routine due to its dynamic imaging capabilities.

In addition to conventional imaging methods, recent advances in artificial intelligence offer promising tools to standardize and potentially improve the objectivity of acromial index measurements. A deep learning approach presented by Selçuk^26^ demonstrated the feasibility of using a YOLOv8-based neural network to automatically extract AI and related parameters from shoulder X-rays, indicating a future direction for integrating machine learning in musculoskeletal imaging. In the future, the application of artificial intelligence in ultrasound imaging may further enhance the precision and reproducibility of mAX measurements. Advances in real-time image analysis and pattern recognition could enable automated detection of anatomical landmarks, minimizing operator dependency. Such AI-assisted sonographic approaches have the potential to establish a standardized and accessible tool for shoulder diagnostics, particularly in resource-limited settings.

In the ultrasound examination in this study, a greater number of pathological joints were identified compared to MRI. This discrepancy may be attributed to the fact that ultrasound allows for a more precise assessment of the maximum extent of the reference points, as supported by previous studies^16,27^. Unlike ultrasound, MRI is a static imaging method, which means that the reference points, when at their maximum extent, might be located between slices or layers. As a result, MRI may not capture the full extent of the pathological changes as effectively as ultrasound. This difference in detection capabilities could account for the variation in findings between the two imaging modalities.

In contrast to the findings of the Bland–Altman analysis, the scatter plot revealed only a moderate inverse correlation between ultrasound-derived mAX and MRI-based AI measurements (r = − 0.595; *p* < 0.001), with a low coefficient of determination (R^2^ = 0.354). This indicates that the relationship between the two modalities lacks consistent linearity across the full measurement range. Although no statistically significant difference was found in the mean values (*p* = 0.237), the evident dispersion and systematic deviation within the scatter plot suggest that the two methods do not produce directly interchangeable results. This divergence between statistical equivalence and graphical variability warrants careful interpretation and underscores the need for caution when considering ultrasound as a standalone modality for AI-based diagnostic decision-making. Moreover, the greater sensitivity of ultrasound to small bony prominences may lead not only to higher detection rates, but also to possible overestimation of pathology. Future studies should clarify whether the observed limits of agreement and measurement differences are clinically acceptable, particularly with regard to decision thresholds for therapeutic interventions.

This study has some limitations: Firstly, the retrospective study design should be considered. A randomized study design might yield different results. Additionally, the study was conducted in a single center with only two investigators, which may limit the generalizability of the findings. A further limitation of the study is that it was conducted exclusively with caucasian participants, which may limit the generalizability of the results to other ethnic groups.

Although the examination of the mAX was performed in standardized sectional planes of the DEGUM and the ultrasound examination is comprehensively trained and routinely applied in German-speaking countries, a possible disadvantage is the different training and application of ultrasound examinations in non-German-speaking countries.

Improved resolution, new technical applications such as ultrasound-assisted 3D imaging of joints, and increased image quality could further enhance the relevance of the sonographic assessment of the mAX in the coming years.

Ma et al. showed that the use of a combination of predictors is better suited to predicting rotator cuff tears than the use of a single parameter alone^28^. In combination with the (ultrasound-) modified critical-shoulder-angle (mCSA) the (ultrasound-)modified acromial index (mAX) is a second parameter which is determined by a quick and radiation-free examination^28^.

Future studies should aim to identify additional predictors that can be determined sonographically. This approach would leverage the benefits of radiation-free examination while combining multiple measurement methods to enhance the accuracy of predicting rotator cuff damage.

## Conclusion

The findings of this study demonstrate that the sonographically assisted determination of the acromial index (mAX) is a reliable, reproducible, and valid method when compared to the MRI-based determination of the standard acromial index. No significant difference was found between the two imaging techniques, indicating that ultrasound can effectively be used as an alternative to MRI for measuring AI.

This study highlights the numerous advantages of ultrasound, including its cost-effectiveness, accessibility, and the absence of ionizing radiation. These benefits are particularly relevant in clinical settings where MRI availability is limited or where quick and dynamic assessments are needed. The dynamic nature of ultrasound allows for more precise identification of anatomical reference points, potentially leading to more accurate measurements.

Despite the promising results, the study acknowledges limitations such as its retrospective design and the single-center setting with only two investigators. These factors may affect the generalizability of the findings. Additionally, differences in ultrasound training and application in non-German-speaking countries could influence the method’s adoption and effectiveness globally.

Future studies should aim to identify additional sonographic predictors to enhance the accuracy of diagnosing shoulder pathologies, leveraging the benefits of radiation-free and cost-effective examinations. Combining multiple ultrasound-based parameters could improve the predictive accuracy for conditions like rotator cuff tears, further establishing ultrasound as a valuable diagnostic tool in orthopedics.

In conclusion, the integration of ultrasound into routine clinical practice for assessing the acromial index can optimize patient care, especially in settings with limited access to advanced imaging modalities. The study supports the broader adoption of ultrasound in shoulder diagnostics, encouraging further research and development in this field.

## Data Availability

The datasets used and/or analysed during the current study available from the corresponding author on reasonable request.
